# Maternal Vaccination in Uganda: Exploring Pregnant Women, Community Leaders and Healthcare Workers’ Perceptions

**DOI:** 10.3390/vaccines9060552

**Published:** 2021-05-25

**Authors:** Phiona Nalubega, Emilie Karafillakis, Lydia Atuhaire, Pamela Akite, Flavia Zalwango, Tracey Chantler, Madeleine Cochet, Janet Seeley, Kirsty Le Doare

**Affiliations:** 1 Medical Research Council/Uganda Virus Research Institute and London School of Hygiene and Tropical Medicine Uganda Research Unit, Entebbe P.O. Box 49, Uganda; phiona.nalubega@mrcuganda.org (P.N.); latuhaire@gmail.com (L.A.); Flavia.Zalwango@mrcuganda.org (F.Z.); Janet.Seeley@LSHTM.ac.uk (J.S.); 2 London School of Hygiene and Tropical Medicine, Keppel Street, London WC1E 7HT, UK; pamela.akite1@alumni.lshtm.ac.uk (P.A.); Tracey.Chantler@lshtm.ac.uk (T.C.); 3 St. George’s, University of London, Cranmer Terrace, London SW17 0RE, UK; mcochet@sgul.ac.uk

**Keywords:** vaccine hesitancy, maternal vaccines, vaccine safety, vaccine confidence

## Abstract

**Background:** We investigated pregnant women, community leaders, healthcare workers (HCWs) and programme managers’ perceptions of maternal vaccination in Kampala, Uganda. **Methods:** We conducted focus group discussions, key informant interviews and in-depth discussions with HCWs (3), community leaders (3), pregnant women (8) and programme managers (10) between November 2019 and October 2020. Data were analysed thematically. **Results:** Pregnant women, community leaders and some HCWs had limited maternal immunisation knowledge. There was confusion over what constitutes a vaccine. Pregnant women may not receive vaccines because of mistrust of government; use of expired vaccines; reliance on traditional medicine; religious beliefs; fear of side effects; HCWs attitudes; and logistical issues. The key facilitators of maternal vaccination were a desire to prevent diseases, positive influences from HCWs and information about vaccine side effects. Community leaders and some pregnant women highlighted that pregnant women do not make decisions about maternal vaccination independently and are influenced by different individuals, including other pregnant women, older people, partners, relatives (parents), community leaders, HCWs and the government. **Conclusions:** Our results indicate that public health messaging should target all community members, including partners and parents of pregnant women as well as HCWs, to improve knowledge of and confidence in maternal vaccines.

## 1. Introduction

Infectious diseases cause morbidity and mortality in the antenatal, postpartum, neonatal and early infant periods [1]. On a global scale, maternal immunisation programmes have made great progress in preventing and reducing infection rates in mother and infant [2]. To maintain these achievements, it is important to ensure that existing and new maternal vaccination programmes are tailored to the contexts in which they are being delivered. This tailoring includes considering beneficiaries’ perceptions and experiences of maternal vaccines and related programme delivery to identify possible concerns that could undermine confidence in vaccination. The importance of contextualised and tailored delivery of vaccination programmes is recognised by the World Health Organization (WHO), noting that vaccine hesitancy is one of the current threats to global health [3]. 

Maternal vaccination has the potential to protect pregnant women and their infants by enabling the transfer of antibodies from mother to child via the placenta and in breastmilk [4]. Currently, the WHO recommends maternal vaccination against tetanus, pertussis, and influenza, as well as other vaccines such as pneumococcus [5].

Tetanus vaccination is the most widely implemented maternal vaccine programme in low- and middle-income countries and the only maternal vaccine officially approved in Uganda [6]. In Uganda, all 15–49-year-old women are offered a schedule of five separate tetanus vaccine doses, starting at age 15 and during their pregnancy (two per pregnancy) in addition to childhood vaccines [7,8]. Recent data from Uganda show that, in 2019, only 65% of pregnant women received two doses of the tetanus vaccine during their pregnancy [9], indicating incomplete coverage and compromised tetanus protection for women and their infants. The 2016 Uganda Demographic and Health Survey also shows a decreased proportion of births protected against neonatal tetanus from 84% in 2011 to 81% in 2016 [10]. 

Currently, there is limited evidence on factors that may influence pregnant women’s acceptance and refusal of maternal vaccination in Uganda that could be used to inform public health programmes [11]. However, studies in other countries report various factors that affect vaccination uptake; for example, in Somalia, concerns about vaccine safety and limited knowledge were mentioned to have affected vaccine uptake among the women [12]. The objective of this study was to develop a better understanding of perceptions around maternal vaccination in Uganda, focusing on the factors that could influence pregnant women’s decisions to accept or refuse vaccination during pregnancy.

## 2. Materials and Methods

We undertook key informant interviews (KIIs), focus group discussions (FGDs) and in-depth interviews (IDIs) between November 2019 and October 2020 to explore HCWs, programme managers, community leaders and pregnant women’s views of the factors influencing maternal vaccination uptake in Uganda.

### 2.1. Study Site

This study was conducted in Kawempe, the largest division in Kampala, the capital city of Uganda, with an estimated population of 338,665 [13]. FGDs and KIIs were conducted in and around the Kawempe division. IDIs and some KIIs were held at the office of the interviewee or in residential areas around the local referral hospital. 

### 2.2. Study Recruitment

Pregnant women, community leaders and HCWs were invited by the research team at Kawempe to take part in the study. Programme managers were invited to take part through emails and phone calls by the social scientists involved in the study. 

HCWs were purposively recruited from the antenatal department, labour suite, immunisation and postnatal departments of the hospital. Programme managers included health workers or staff with vaccination managerial roles within the Ministry of Health and Kampala government health facilities. Community leaders from the villages surrounding the hospital were identified and selected to include a mix of religious leaders, community leaders, village health teams, traditional birth attendants and women leaders. Pregnant women at different stages of pregnancy were recruited from the antenatal department at the hospital. Women who took part in FGDs were divided into three groups: younger mothers (17–24 years), more mature women (>25 years) and women with more than three pregnancies.

### 2.3. Topic Guides and Data Collection

Semi-structured topic guides were designed to obtain the participants’ sociodemographic information, as well as information on the participants’ vaccine awareness and vaccination experiences and their views on the factors that influence maternal vaccination, including influencers that shape pregnant women’s decision to be vaccinated.

After providing written informed consent, participants took part in FGDs, KIIs or IDIs, conducted by experienced qualitative interviewers, lasting between 60 and 120 min. For the non-literate participants, friends and partners served as witnesses of the consenting process. All interviews took place in a private setting that was safe for both participants and researchers. Interviews were conducted in English and in Luganda for participants who did not understand English. Pregnant women were selected on the basis of age and parity. The HCWs, community leaders and programme managers were selected based on their roles in the community and maternal vaccination.

### 2.4. Data Management and Analysis

All FGDs, IDIs and KIIs were audio-recorded, transcribed verbatim into Microsoft Word, and if conducted in Luganda, translated into English. The transcripts were cross-checked against the audio recordings by the research team to ensure accuracy.

A coding framework was developed by P.N., F.Z., L.A., P.A. and E.K. based on the questions included in the topic guide, the study objectives and pilot coding of two interviews. Anonymised transcripts were coded manually by PN using the coding framework in Excel. Data were analysed thematically, drawing on themes based on the research objectives related to vaccine perceptions used in the interview guide as well as those emerging from the data. The analysis was an iterative process of discussion and revision among co-authors. 

## 3. Results

A total of 135 participants took part in this study, including 28 HCWs divided into three FGDs, 10 programme managers for KIIs, 25 community leaders divided into three FGDs and 72 pregnant women, 12 of whom took part in IDIs and 60 took part in 8 FGDs. 

The following three key themes were identified during analysis: (1) awareness of maternal vaccines, (2) facilitators and deterrents of maternal vaccination, and (3) the role of influencers in decision-making. They are described in further detail below.

### 3.1. Awareness of Maternal Vaccines

While some HCWs, programme managers and pregnant women demonstrated an awareness of available maternal vaccines (tetanus toxoid/tetanus diphtheria), a few community leaders and most pregnant women referred to different types of treatments given during antenatal care (ANC), such as malaria prophylaxis/treatment (e.g., Fansidar) and antiretroviral treatment for HIV as vaccines. 

When asked about maternal vaccination, HCWs and programme managers also referred to Anti-D (a prescription medication used to prevent rhesus immunisation, also known as rhesus incompatibility) and magnesium sulphate, as well as vaccines not currently given during pregnancy such as those against hepatitis B and rubella.

*“We have another vaccine we give to women and it is called Anti-D. It is mostly given to women who are rhesus negative for example when they have ‘A’ negative. Therefore such a woman is given an Anti-D vaccine to prevent the baby from dying.”*

(FGD1, Healthcare workers)

### 3.2. Perceived Facilitators and Barriers or Deterrents to Maternal Vaccination 

#### 3.2.1. Barriers to Maternal Vaccination 

Participants mentioned various factors likely to hinder pregnant women from receiving maternal vaccines. 

#### 3.2.2. Rumours and Conspiracy Theories

Some pregnant women discussed women’s mistrust in relation to the use of expired vaccines which “*affect the baby’s brain*” (pregnant women’s FGD5), which affected perceptions around the safety of vaccines.

Conspiracy theories were also perceived by HCWs and programme managers as an important factor that could hinder maternal vaccine uptake. They described women who believed that there was a hidden agenda related to population control in the provision of maternal vaccines:

*“There is a myth where people think that maybe government put some harmful things in the vaccines. That is why they even refuse their children from being immunised. In the village, they think that if you take those vaccines, you can die.”*

(FGD2, Healthcare workers)

*“They say that the ‘Bazungu’ [whites] want to kill them. They say that the Bazungu have their tricks and that is the way they want to reduce the [number of] Africans [by causing infertility].”*

(FGD2, Healthcare workers)

In addition to these rumours, apprehension around vaccine safety was also expressed. All participants believed that a strong deterrent to maternal vaccination is some women’s belief that vaccines may cause side effects:

*“Currently, if a pregnant woman gives birth to a child, they get jaundice. Some people think that the vaccines they use to vaccinate pregnant women are the ones that cause such illnesses like jaundice. That is why most people don’t want to go to the hospital to get that western medicine [vaccines] because of the fear of side effects.”*

(FGD1, Community leaders)

*“They say that some of these vaccines make their baby boys infertile. So they tell us that we should not step at their doorstep [to mobilise them for vaccination].”*

(FGD2, Healthcare workers)

*“Some people think that vaccines make them have stillbirths.”*

(FGD2, Community leaders)

Pregnant women were perceived to trust in local remedies, which they might use instead of Western medicines or vaccination.

*“In most cases people in the communities trust their local herbs and they are the ones they mostly use so that is why some people take them and even fail to go to the hospital to attend antenatal [care].”*

(IDI2, Pregnant woman)

HCWs and pregnant women also believed that fear of pain or injections could affect uptake of maternal vaccines by pregnant women.

*“Most women just fear the pain of the injection. Even if you tell them the benefits of the vaccines, they boldly tell you that the injection is so painful.”*

(FGD1, Healthcare workers)

##### The Role of Religion

Religious beliefs were perceived by HCWs, community leaders, pregnant women and programme managers as a factor that could deter maternal vaccination uptake. Some religious groups were said not to accept certain medications, such as Jehovah’s witnesses, some Pentecostal Christians and Tabliqs (a Muslim group). The participants explained that members of these groups believed that certain medications are associated with satanic practices and receiving them would be disrespectful to their God.

*“There is also my neighbour who usually tells me that I shouldn’t get vaccinated because health workers don’t work better than God. My neighbour is a ‘born-again’ [Pentecostal] and she usually says that God is better than all those health workers.”*

(FGD2, Pregnant women)

In addition to such beliefs and concerns, there were also practical issues that hindered uptake.

##### Time and Logistics

Distance and lack of transport to the vaccination site affected pregnant women’s ability to access vaccination.

*“Sometimes the long distances they have to travel to go to the hospital to get vaccinated hinder them. One may be staying here [suburbs] yet the hospital where they vaccinate from is as far as Kampala [City Centre] and if the woman doesn’t have transport, she may lack the energy to walk up to the hospital.”*

(FGD1, Community leaders)

Some pregnant women also reported time and logistic barriers related to their work as something that could deter them from getting vaccinated during pregnancy. For example, one woman was unable to get vaccinated because she could not leave work:

*“I am a teacher and it is very hard to be permitted to get leave at the school where I am working. I even get difficulties taking my children for immunisation. […] If you ask for a leave day at school, our bosses feel like it is burden for them. If you take like three days off at school, they might even reduce on your salary. I usually start attending antenatal at five months so that I visit the hospital like three times instead of starting early and having to visit the hospital several times.”*

(FGD6, Pregnant women)

Other pregnant women reported long queues at the facility as something that could deter maternal vaccination. They reported that there are usually many pregnant women waiting at the facilities to receive vaccination services.

*“One may say ‘the line is too long for me to wait yet I have things to do’. So such a woman ends up not getting vaccinated. [She] may come back the second time at the hospital and the moment she realises that the line is too long, she just goes back without getting vaccinated.”*

(IDI3, Pregnant woman)

Being late for antenatal visits was also reported as a deterrent to maternal vaccination by some pregnant women. Since vaccination for women is integrated into antenatal services, some women who report late for antenatal care end up missing these services. 

HCWs’ attitudes and the way they interact with pregnant women were also reported by community leaders as one factor that could hinder maternal vaccination.

*“Health workers should not be tough before those women. That is one of the things that makes women refuse to get vaccinated. They fear going to the hospital because the health workers abuse them and sometimes shout at them.”*

(FGD3, Community leaders)

### 3.3. Facilitators for Maternal Vaccine Uptake

When participants were asked about what they thought could facilitate maternal vaccination uptake and acceptance, they mentioned several factors, including positive influence from HCWs, perceptions about the tolerability of the vaccine, access to vaccination, as well as the positive influences from partners or relatives.

HCWs and programme managers thought that pregnant women may be more likely to accept vaccination if they are actively motivated by HCWs. They explained that since pregnant women are usually asked about their ANC attendance around the time of delivery, pregnant women think that a failure to attend such sessions may mean that they will get less attention from HCWs who believe they may have missed appointments, including vaccination.

*“The women go for ANC with their different reasons. […] they know that the midwives will harass them if they don’t have a TT [Tetanus vaccine] or antenatal card.”*

(KII3, Programme manager)

Pregnant women perceived that receiving information on vaccine side effects could facilitate maternal vaccine uptake, with women more willing to accept vaccines that are “tolerable” or with fewer side effects.

*“Apart from the pain, so far Td is tolerable. Uptake would be bad if the vaccine administered in pregnancy is hard to tolerate as a pregnant woman. Apart from the arm being paralysed for the next two days, it is tolerable. At least that is the vaccine I have known which is being administered among the pregnant women. The vaccine not being tolerated would affect the uptake. The current vaccine is tolerable.”*

(KII9, Pregnant woman and programme manager)

Being knowledgeable about the benefits of maternal vaccines also influenced the decision to vaccinate, with some pregnant women accepting vaccination during pregnancy to protect themselves and their babies from getting infections.

### 3.4. Role of the Community in Influencing Vaccination Decisions

Community leaders and some pregnant women explained that pregnant women do not make decisions about maternal vaccination by themselves and are influenced by others, including other pregnant women, older people in the community, their partners, relatives (particularly their parents), community leaders, HCWs and the government.

*“Some women tell their fellow women that the injection for the tetanus vaccine is very painful and those who have never had that vaccine get scared and don’t get vaccinated.”*

(FGD1, Community leaders)

Older people were perceived by community leaders and pregnant women as possible influencers in pregnant women’s decision to vaccinate and sometimes make decisions for them. Community leaders explained that as vaccinations were not available for pregnant women in the past (tetanus vaccination was recommended in pregnancy by the WHO in 1998) and children were seen as growing up to be healthy, older generations believed that vaccines were not necessary for pregnant women.

*“[…] grandparents or people who are older than us […] say that they gave birth to our mothers when they had not been vaccinated. So, the women get influenced by those older people and start saying ‘if my grandparent gave birth without getting vaccinated, I will also refuse to get vaccinated.’”*

(FGD3, Pregnant women)

*“Some older women discourage us from receiving those vaccines since some are expired; they usually tell us that those vaccines can make a woman give birth to a child with a disability.”*

(FGD2, Pregnant women)

Community leaders said that a woman’s partner could influence the decision to vaccinate. They explained that in some homes, men’s decisions are considered more important due to the patriarchal structure of most families, with women not allowed to make decisions before consulting their husbands. If men refuse vaccination for their wives, pregnant women will follow their husband’s decision.

Community leaders were also believed to play an important role in influencing women to make decisions about maternal vaccination. Community leaders such as Village Health Teams (the lowest tier of the government health service), local council and religious leaders were perceived by community leaders as a group of people that influence women’s decision to vaccinate because pregnant women trust what they say. Therefore, if community leaders do not accept any kind of medication, it was perceived that the people they lead are most likely to follow what they do.

Community leaders said that the role of influencers on decision-making can be limited by governments mandating certain interventions, such as maternal vaccination. They explained that some women may accept vaccination, because they believe it is a government requirement:

*“Whether you accept or not, the government can force you to get vaccinated. If they make it a must for everyone to get vaccinated as they recently did to the children who were in school, you must get vaccinated. We even saw some parents being taken to police for refusing to get their children vaccinated. If something is beneficial to the mother and the baby, the government can make a decision for us.”*

(FGD3, Community leaders)

However, some pregnant women believed that they were the ones responsible for making their own decisions about vaccination. The consensus among women was that the decision to receive maternal vaccines is made by the pregnant woman, regardless of other influencers such as relatives, older women and community members. 

## 4. Discussion

We have described pregnant women, community leaders, HCWs and programme managers’ perceptions of the barriers to and enablers of maternal vaccination and the role of community and family in decision-making in an urban Ugandan setting.

Despite tetanus vaccines being the only approved vaccines in Uganda, participants described other ANC treatments given to women as “vaccines”, which could imply an important knowledge gap. This could be due to the fact that tetanus toxoid vaccination during pregnancy is integrated into antenatal care [10], leading to participants’ confusion and difficulty in differentiating between interventions or, as we found, due to the perception that all medication that protects against infection is a “vaccination”. A study in Eastern Uganda also identified issues with women describing antimalarial medication as a vaccine, which highlights the need for future public information campaigns to focus on the meaning of vaccination [11].

Globally, concerns regarding maternal vaccine safety is one of the major barriers to vaccination during pregnancy [14] and this is reflected in our results, where safety concerns might affect the decision to vaccinate. This is also true of other African countries, such as Kenya and South Africa, where one of the main barriers to maternal vaccination is vaccine safety concerns and fear of side effects [14,15,16].

Trust in the intention of global health bodies and governments in recommending vaccination is critical for uptake of maternal, as well as other vaccine services [17,18]. In recent years the use of social media has encouraged the sharing of mis-information about vaccines [19], but, as shown in our study, rumours fuelled by fears over unwanted external influence have long undermined trust in vaccination programmes and the health workers providing the vaccinations [18,20]. The increase in antivaccination movements in Uganda and internationally can only be tackled with sustained dialogue with the communities receiving immunisation to improve vaccine confidence. While mandating vaccines or imposing penalties for non-vaccination has been proposed as a strategy to increase vaccination uptake, such strategies can also entail important ethical challenges, especially if they are not implemented together with tailored information materials and complemented by community engagement strategies to improve trust in vaccination [19].

The effect of distance on the utilisation of health services in settings increases when it is combined with lack of transportation [21]. Our findings concur with other research reporting distance to service delivery points as one of the major factors leading to low vaccination coverage in Uganda [22]. Time taken to attend antenatal care is also highlighted as a potential barrier to vaccine uptake, indicating that this is a key area of capacity strengthening required in vaccination programmes in pregnancy [23].

HCW attitudes are reported to hinder pregnant women from receiving antenatal care in Kenya and South Africa, similar to our findings, thus affecting maternal vaccination uptake [24,25]. It is important to consider the training implications of positive attitudes of HCW towards women and their messaging on vaccination to ensure that high antenatal vaccination coverage is maintained.

The influence of partners on maternal vaccinations uptake and acceptance during pregnancy was highlighted in our study. Other research in Uganda has shown the important role of male partners in maternal decision-making about childhood vaccination [26], and male partners were found to act as strong influencers on pregnant women’s overall health and their access to care in South Africa [27]. A literature review that was conducted to understand the factors that contributed to vaccine uptake among pregnant women also presented five articles where pregnant women’s husbands significantly affected their likelihood to be vaccinated [28]. 

Our study highlighted the importance of religious beliefs in the decision to vaccinate. Uganda has different religious groups, which do not encourage their followers to engage in vaccination services, who believe that their God cures every kind of illness and that vaccinations are therefore not necessary [22]. A study of infant vaccination hesitancy in Uganda also identified religious beliefs as one of the barriers to effective uptake and provision of immunisation services [22] and similar results were found in Zambia [29]. Taken together, these findings indicate the importance of considering the wider community in vaccine decision-making for pregnant women. 

Regardless of the findings from our study, which showed different factors that hinder women from getting vaccinated, there are no laws imposed on whoever rejects the vaccines in Uganda, yet one study conducted in Europe reported some legal enforcements that encourage people to take up vaccines [20].

### Limitations

There are several limitations to our study. We did not include interviews with the partners of pregnant women, which could have provided important findings, as male partners are important influencers in health and vaccination decisions in households in Uganda. Furthermore, the study was only conducted in an urban setting and may not be applicable to other settings such as rural areas. Our findings relate to a group of women in Kampala, and, although many of our findings are similar to other Ugandan studies, they may not be similar outside the country. 

## 5. Conclusions

Our study findings show that knowledge of maternal vaccines and perceptions about their use are limited among some pregnant women and others who may influence vaccine uptake, suggesting the need for increased communication and engagement strategies around maternal vaccination. We suggest that public health messaging target whole communities and advocate for the co-creation of materials to make sure that the message is relevant to all those making decisions about vaccination during pregnancy. The findings from this study can inform policy makers, policy analysts and vaccination programme managers to support the design of appropriate maternal vaccination programmes and public health campaigns, taking into consideration the role of the different influencers that play a key role in decision-making.

## Data Availability

There is no additional data available for this study.
